# Pruritus Treated with Vinegar

**Published:** 1876-01

**Authors:** 


Pruritus Treated with Vinegar.—Dr. Thackeray, of Dakota, writes to the *Medical News* that he treats the pruritus formicans of pregnancy with cider vinegar, topically applied, and that he has never failed to cure with it. He procured the prescription from the late Professor S. Henry Dickson.—*Pacific Med & Surg. Jour.*

